# Feature-Based Molecular Networking for the Exploration of the Metabolome Diversity of Common Egyptian *Centaurea* Species in Relation to Their Cytotoxic Activity

**DOI:** 10.3390/molecules28020674

**Published:** 2023-01-09

**Authors:** Eman H. Reda, Nesrine M. Hegazi, Mona Marzouk, Zienab T. Abdel Shakour, Ali M. El-Halawany, El-Sayeda A. El-Kashoury, Tarik A. Mohamed, Mahmoud A. A. Ibrahim, Khaled A. Shams, Nahla S. Abdel-Azim, Christopher J. Kampf, Thomas Efferth, Paul. W. Paré, Mohamed-Elamir F. Hegazy

**Affiliations:** 1 Phytochemistry Laboratory, National Organization for Drug Control and Research, Giza 12622, Egypt; 2 Phytochemistry and Plant Systematics Department, National Research Centre, Dokki, Cairo 12622, Egypt; 3 Department of Pharmacognosy, Faculty of Pharmacy, Cairo University, Cairo 11562, Egypt; 4 Chemistry of Medicinal Plants Department, National Research Centre, 33 El-Bohouth St., Dokki, Giza 12622, Egypt; 5 Computational Chemistry Laboratory, Chemistry Department, Faculty of Science, Minia University, Minia 61519, Egypt; 6 School of Health Sciences, University of Kwa-Zulu-Natal, Westville, Durban 4000, South Africa; 7 Department for Chemistry, Johannes Gutenberg University Mainz, Duesbergweg 10-14, 55128 Mainz, Germany; 8 Department of Pharmaceutical Biology, Institute of Pharmaceutical and Biomedical Sciences Chemistry, Johannes Gutenberg University, Staudinger Weg 5, 55128 Mainz, Germany; 9 Department of Chemistry & Biochemistry, Texas Tech University, Lubbock, TX 79409, USA

**Keywords:** *Centaurea*, cytotoxicity, LC-MS/MS, molecular networking, sesquiterpene lactones

## Abstract

*Centaurea* is a genus compromising over 250 herbaceous flowering species and is used traditionally to treat several ailments. Among the Egyptian *Centaurea* species, *C. lipii* was reported to be cytotoxic against multidrug-resistant cancer cells. In this context, we aimed to explore the metabolome of *C. lipii* and compare it to other members of the genus in pursuance of identifying its bioactive principles. An LC-MS/MS analysis approach synchronized with feature-based molecular networks was adopted to offer a holistic overview of the metabolome diversity of the Egyptian *Centaurea* species. The studied plants included *C. alexandrina, C. calcitrapa, C. eryngioides, C. glomerata, C. lipii, C. pallescens, C. pumilio,* and *C. scoparia*. Their constitutive metabolome showed diverse chemical classes such as cinnamic acids, sesquiterpene lactones, flavonoids, and lignans. Linking the recorded metabolome to the previously reported cytotoxicity identified sesquiterpene lactones as the major contributors to this activity. To confirm our findings, bioassay-guided fractionation of *C. lipii* was adopted and led to the isolation of the sesquiterpene lactone cynaropicrin with an IC_50_ of 1.817 µM against the CCRF-CEM leukemia cell line. The adopted methodology highlighted the uniqueness of the constitutive metabolome of *C. lipii* and determined the sesquiterpene lactones to be the responsible cytotoxic metabolites.

## 1. Introduction

*Centaurea* is the fourth largest genus in the Asteraceae family [1] and has approximately 250 species (400 in an earlier classification) that are mostly centered in the Mediterranean region. The genus includes diverse biologically active metabolites, including sesquiterpene lactones, triterpenes, flavonoids, and lignans [2]. Owing to such metabolic diversity, many biological activities were reported for its members such as anti-inflammatory, antimicrobial, antioxidant, hepatoprotective, etc. [3].

Traditionally, *Centaurea* species have long been used to cure several disorders, including liver diseases, the common cold, diabetes, and malaria [4]. A variety of *Centaurea* species are also prescribed as herbal remedies against inflammatory conditions, such as abscesses, asthma, hemorrhoids, peptic ulcers, malaria, common colds, and abdominal pain [5].

With an escalating demand for anticancer drugs to combat multidrug-resistant tumors, re-exploring our natural resources for potential anticancer agents is warranted. This is especially true since only a small proportion of plants have specifically been assayed for antitumor activity. Following the primary objective of our group, which is to investigate our natural resources for the discovery of potentially bioactive secondary metabolites [6,7,8], Egyptian *Centaurea* species were previously screened for their cytotoxic potential toward multidrug-resistant cancer cells. The results of the study revealed the superiority of *C. lipii* in reducing the cell viability to less than 20% at a concentration of 10 μg/mL [9].

Such findings suggested the necessity of exploring the metabolome of *C. lipii* and highlighting the metabolome differences in the regionally specific *Centaurea* species. Accordingly, state-of-the-art metabolomic tools were exploited to map the metabolome diversity of eight *Centaurea* species (*C. alexandrina*, *C. calcitrapa*, *C. eryngioides*, *C. glomerata*, *C. lipii*, *C. pallescens*, *C. pumilio*, and *C. scoparia*) in the context of their formerly reported cytotoxic activity against multidrug-resistant cancer cells. The adopted approach comprised LC-MS/MS analysis combined with spectral similarity networks through the Global Natural Products Social Molecular Networking (GNPS) platform. The recorded metabolome was linked to the previously documented cytotoxic activity to highlight the potential bioactive metabolites. This was followed by bioactivity-guided isolation of the cytotoxic metabolites from *C. lipii* to validate our findings.

## 2. Results

### 2.1. Comparative Analysis of LC-MS/MS Profiles from Centaurea Species

The LC-MS/MS analysis of the selected *Centaurea* extracts showed clear qualitative and quantitative differences as observed in their respective base peak chromatograms (Supplementary Figure S1).

As *C. lipii* was the species with the most potent cytotoxicity, as indicated in a previous screening of Egyptian plant extracts [9], the identification of the contributing metabolites was our goal. Accordingly, a feature-based molecular network was constructed to better visualize the metabolome diversity of the selected *Centaurea* species and to accentuate the uniqueness of *C. lipii*.

### 2.2. MS/MS Molecular-Networking-Based Phytochemical Investigations

**Figure 1:**
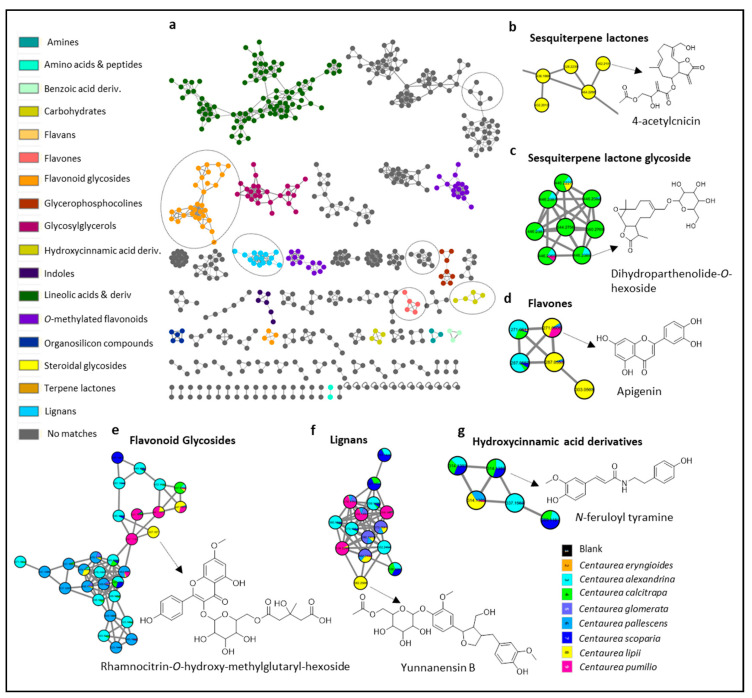
Metabolome profiling of 8 Egyptian *Centaurea* species and relative distribution of the metabolites among the studied species. (**a**) Enhanced molecular network of the ESI-positive MS/MS spectra using MolNetEnhancer showing different molecular families/clusters of the pooled metabolites in the studied species. Node colors represent classes of putatively annotated metabolites with matches found in the GNPS libraries. (**b**–**g**) Clusters of the different metabolite classes, shown as pie charts illustrating their distribution in the studied *Centaurea* species.

For a global overview, feature-based molecular networking (FBMN) was applied for the visual exploration of the discrepancy in the recorded metabolome of the studied species as well as for facilitating the metabolite annotation. The constructed FBMN was then analyzed using a MolNetEnhancer workflow which enhances the data annotation via combining outputs from different computational tools [10] (Figure 1a).

The constructed MN consisted of 977 nodes grouped in 77 clusters (with a minimum of 2 connected nodes) and 385 single nodes (Figure 1). Then, the node and edge attributes were employed so that the color of a node corresponded to the name of the studied *Centaurea* species. The nodes are displayed as a pie chart to reflect the distribution of each ion among the 8 species (Figure 1).

The recorded metabolome encompassed unidentified clusters with no matches which were manually inspected and identified (i.e., clusters b and c corresponded to the sesquiterpene lactones, Figure 1b) which could be explained by their presence as either ammonia [M+NH_4_]^+^ or acetonitrile [M+ C_2_H_3_N +H]^+^ adducts. 

The clusters of interest were as follows: cluster **b**: sesquiterpene lactones, cluster **c**: sesquiterpene lactone glycosides, cluster **d**: flavones, cluster **e**: flavonoid glycosides, cluster **f**: lignans, and cluster **g**: hydroxycinnamic acid derivatives (Figure 1b–g).

**Figure 2:**
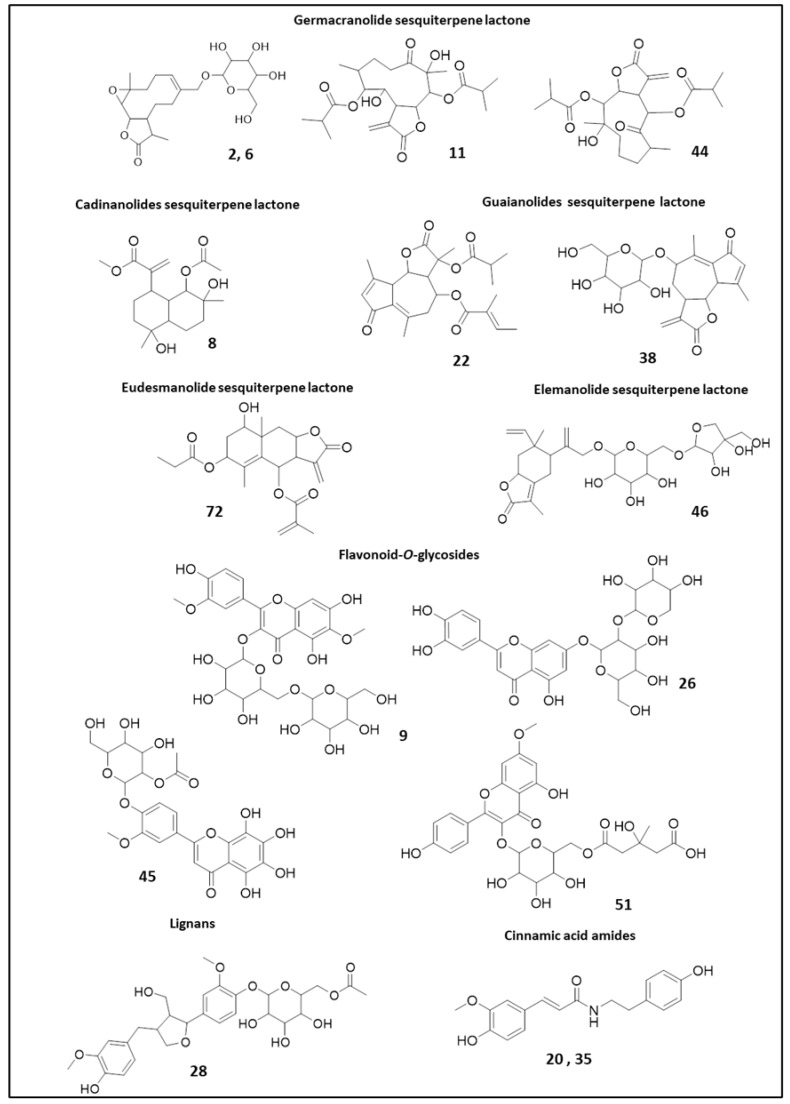
Representative examples of compounds reported in the *Centaurea* genus for the first time.

As delineated in Figure 1b and Supplementary Table S1, a total of 81 metabolites were tentatively assigned belonging to different chemical classes. This included 49 flavonoids, 15 sesquiterpene lactones, 10 lignans, 4 cinnamic acid derivatives, and 2 coumarins. Figure 2 displays representative examples of the compounds reported here in the genus *Centaurea* for the first time, and following is a discussion of the annotated metabolites in their elution order.

#### 2.2.1. Hydroxycinnamic Acid Derivatives

Hydroxycinnamic acid derivatives were observed in the constructed FBMN (Figure 1g) exclusively as ferulic acid derivatives as confirmed by their shared daughter ions at *m/z* 177 and 145. This included feruloyl quinic acid ester (**1**, *m/z* 369.1179 [M+H]^+^, C_19_H_20_O_9_) previously reported to occur in *Centaurea* [11], followed by its amide derivatives as *N*-feruloyl tyramine isomers (**20** and **25**, *m/z* 314.1388 [M+H]^+^, C_18_H_19_NO_4_), and *N*-feruloyl tryptamine (**66**, *m/z* 337.1563 [M+H]^+^, C_20_H_20_N_2_O_3_) not formerly reported in *Centaurea*.

#### 2.2.2. Sesquiterpene Lactones

Unlike the cinnamic acid derivatives which showed no significant difference in distribution among *Centaurea* species, sesquiterpene lactones showed a different pattern. Sesquiterpene lactones were almost exclusively detected in *C. lipii* with few occurring in *C. calcitrapa* and *C. eryngioides*.

**Figure 3:**
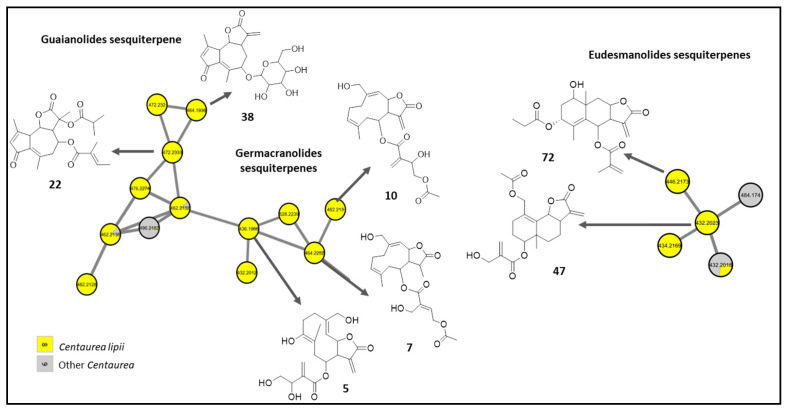
Examples of sesquiterpene lactones exclusively found in *C. lipii*.

Sesquiterpene lactones are a group of secondary metabolites widely distributed in the Asteraceae family and are classified according to their carbocyclic skeletons into different classes, i.e., germacranolides, eudesmanolides, guaianolides, and pseudoguaianolides. Several sesquiterpene lactones were detected exclusively in *C. lipii*, belonging to the germacranolides, guaianolides, cadinanolides, elemanolides, and eudesmanolides (Figure 3). Annotated sesquiterpene lactones were detected as adducts of acetonitrile [M+C_2_H_3_N+H]^+^ while glycosidic derivatives were seen as ammonia adducts [M+NH_4_]^+^ (Supplementary Table S1). Interestingly, the annotated sesquiterpene lactones were mostly reported previously in the genus except for the glycosidic ones which are reported here for the first time in *Centaurea*.

Among the annotated sesquiterpene lactones, germacranolides were the most abundant class. Germacranolide glycosides were tentatively assigned as dihydroparthenolide-*O*-hexoside isomers (**2** and **6**, *m/z* 446.2385 [M+NH_4_]^+^, C_21_H_32_O_9_), particularly in *C. calcitrapa* and *C. eryngioides*, and described for the first time in *Centaurea*. 

The nonglycosidic ones were found mainly in *C. lipii* and included 7-hydroxy-10-(hydroxymethyl)-6-methyl-3-methylidene-2-oxo--cyclodeca[b]furan-4-y-3,4-dihydroxy-2-methylidenebutanoate (**5**, *m/z* 436.1966 [M+C_2_H_3_N+H]^+^, C_20_H_26_O_8_), 10-(hydroxymethyl)-3,6-dimethyl-2-oxo-cyclodeca[b]furan-4-yl-4-(acetyloxy)-2-(hydroxymethyl)but-2-enoate (**7**, *m/z* 464.2252 [M+C_2_H_3_N+H]^+^, C_22_H_30_O_8_), 4-acetylcnicin (**10**, *m/z* 462.1227 [M+C_2_H_3_N+H]^+^, C_22_H_28_O_8_), incaspitolide D (**11**, *m/z* 496.2529 [M+C_2_H_3_N+H]^+^, C_23_H_34_O_9_), and incaspitolide A (**44**, *m/z* 480.2583 [M+C_2_H_3_N+H]^+^, C_23_H_34_O_8_).

A previous study reported the presence of **10** in *C. calcitrapa* collected in Spain [12], but it was not detected in the same species included in this study which suggests the possible effect of geographical factors on the chemical profiles of this species.

Similarly, a guaianolide glycoside dehydrolactuside C (**38**, *m/z* 464.1936 [M+C_2_H_3_N+H]^+^, C_21_H_26_O_9_) and the elemanolide glycoside sarcaglaboside D (**46**, *m/z* 560.2703 [M+NH_4_]^+^, C_26_H_38_O_12_) were detected. Nonglycosidic guaianolides were described as daucoguaianolactone F (**22**, *m/z* 472.2333 [M+C_2_H_3_N+H]^+^, C_24_H_30_O_7_), 8-(acetyloxy)-9-hydroxy-9-(hydroxymethyl)-3,6-dimethylidene-2-oxo-octahydroazuleno [4,5-b]furan-4-yl 2-(hydroxymethyl)prop-2- enoate (**23**, *m/z* 432.2012 [M+C_2_H_3_N+H]^+^, C_21_H_26_O_7_), and clementein (**47**, *m/z* 432.2023 [M+C_2_H_3_N+H]^+^, C_21_H_26_O_7_). Though no reports exist for the bioactivity of **22**, metabolites with a daucoguaianolactone group were reported to possess cytotoxic activity [13]. Thus, this compound may contribute positively to the cytotoxicity of *C. lipii* [9].

Besides the observed germacranolides and guaianolides, the cadinanolide acetoxy-dihydroxy-tetrahydroartemisinic acid methyl ester (**8**, *m/z* 382.2220 [M+C_2_H_3_N+H]^+^, C_18_H_28_O_6_) and the eudesmanolide propyloxy-methyl-cryloxyivangustin (**72**, *m/z* 446.2173 [M+C_2_H_3_N+H]^+^, C_22_H_28_O_7_) were observed.

#### 2.2.3. Flavonoids

Similar to the bioactive sesquiterpene lactones discussed earlier, *Centaurea* species are well known for their high content of flavonoids [14].

In our investigation, methylated flavonols and flavones were the predominant species, occurring as diglycosides, monoglycosides, acylated monoglycosides, or as free aglycones. In total, 49 flavonoids were annotated, some of which were previously reported to exist in the genus (Supplementary Table S1). The detected flavonoids showed the typical fragmentation sequence of *O*-glycosidic flavonoids of the loss of 162, 146, or 132 corresponding to O-hexoside, *O*-deoxyhexoside, or *O*-pentoside, respectively.

Considering the studied *Centaurea* species, flavonoid glycosides were among the most abundant metabolite class appearing as cluster **e** in the constructed MN (Figure 1e). Among the annotated flavonoids, methoxylated flavones and flavonols occurred mainly as monoglycosides, agreeing with the literature. Among the annotated flavonoid glycosides were isomers of patuletin-*O*-glucoside (**13**, **15,** and **17**, *m/z* 495.1129 [M+H]^+^, C_22_H_22_O_13_), luteolin-7-*O*-rutinoside (**16**, *m/z* 595.1663 [M+H]^+^, C_27_H_30_O_15_), isorhamnetin-*O*-glucoside (**19**, *m/z* 479.1185 [M+H]^+^, C_22_H_22_O_12_), luteolin-7-*O*-glucoside (**18**, *m/z* 449.1082 [M+H]^+^, C_21_H_20_O_11_), isomers of hispidulin-7-*O*-glucuronide (**24** and **30**, *m/z* 477.1029 [M+H]^+^, C_22_H_20_O_12_), isomers of spinacetin -*O*-glucoside (**25** and **40**, *m/z* 509.1289 [M+H]^+^, C_23_H_24_O_13_), isomers of isorhamnetin-*O*-glucoside (**27** and **37**, *m/z* 479.1185 [M+H]^+^, C_22_H_22_O_12_), isomers of apigenin-*O*-glucuronide (31 and **33**, *m/z* 447.0927 [M+H^]+^, C_21_H_18_O_11_), isomers of monomethoxy trihydroxyflavone-*O*-glucoside (**34** and **42**, 463.1240 [M+H]^+^, C_22_H_22_O_11_), apigenin-*O*-hexoside (**36**, *m/z* 433.1132 [M+H]^+^, C_21_H_20_O_10_), isomers of trihydroxy-dimethoxy-flavone-*O*-glucoside (**41** and **48**, *m/z* 493.1342 [M+H]^+^, C_23_H_24_O_12_), isomers of trimethoxy trihydroxyflavone-*O*-glucoside (**43** and **49**, *m/z* 523.1451 [M+H]^+^, C_24_H_26_O_13_), along with the apigenin-*O*-methyl glucuronide (**52**, *m/z* 461.1077 [M+H]^+^, C_22_H_20_O_11_) which were previously reported in *Centaurea* species. Aside from the aforementioned flavonoid-*O*-glycosides, one *C*-glycosidic flavonoid was annotated as (iso)vitexin (**12**, *m/z* 433.1135 [M+H]^+^, C_21_H_20_O_10_).

Likewise, the monoglycosides previously mentioned, the diglycosides are reported here for the first time in *Centaurea,* i.e., spinacetin-*O*-gentiobioside (**9**, *m/z* 671.18 [M+H]^+^, C_29_H_34_O_18_) and luteolin-O-pentosyl-*O*-hexoside (**26**, *m/z* 581.1502 [M+H]^+^, C_26_H_28_O_15_). Similarly, the acylated flavonoid glycosides were not previously described, i.e., pentahydroxy-monomethoxy flavone-*O*-acetyl hexoside (**45**, *m/z* 537.1239 [M+H]^+^, C_24_H_24_O_14_), rhamnocitrin-*O*-hydroxy-methylglutaryl-hexoside (**51**, *m/z* 607.1670 [M+H]^+^, C_28_H_30_O_15_), luteolin-*O*-acetyl hexoside (**56**, *m/z* 491.1192 [M+H]^+^, C_23_H_22_O_12_), isorhamnetin -*O*-acetyl hexoside (**57**, *m/z* 521.1291 [M+H]^+^, C_24_H_24_O_13_), and syringetin *O*-acetyl hexoside (**58**, *m/z* 551.1389 [M+H]^+^, C_25_H_26_O_14_).

Lastly, 21 flavonoid aglycones were described in this study (Supplementary Table S1). Regarding the distribution of the flavonoids, no specific pattern was observed except a higher prevalence in *C. alexandrina* and *C. pallescens* (Figure 1e).

#### 2.2.4. Lignans

In addition to sesquiterpene lactones and flavonoids, *Centaurea* is known to produce lignans [15], mainly as the dibenzylbutyrolactone type. Reported lignans include matairesinol and arctigenin along with their glycosides matairesionoside and arctiin, which were reported to exert anticancer effects against colorectal cancer [16].

In our study, lignan glycosides existed as ammonia adducts [M+NH_4_]^+^ as commonly detected in the positive ionization mode used [17]. Annotated lignans included previously reported ones such as matairesinol-*O*-glucoside (**14**, *m/z* 538.2286 [M+NH_4_]^+^, C_26_H_32_O_11_), isomers of arctigenin-*O*-glucoside (**21**, **29,** and **39**, *m/z* 552.2282 [M+NH_4_]^+^, C_27_H_34_O_11_), matairesinol (**50**, *m/z* 359.1496 [M+H]^+^, C_20_H_22_O_6_), isomers of arctigenin (**53** and **59**, *m/z* 373.1637 [M+H]^+^, C_21_H_24_O_6_), and [(dimethoxyphenyl)methyl]-3-[(hydroxy-methoxyphenyl) methyl]-tetrahydrofuranone (**55**, *m/z* 390.1914 [M+NH_4_]^+^, C_21_H_27_O_6_).

Additionally, the occurrence of secoisolariciresinol (**32**, *m/z* 327.1594 [M+H]^+^, C_20_H_22_O_4_) in *Centaurea* is reported here for the first time together with the acetylated lignan glycosides exemplified by acetyl matairesinoside (**28**, *m/z* 552.2438 [M+NH_4_]^+^, C_27_H_34_O_11_) occurring exclusively in *C. lipii*.

### 2.3. Bioactivity-Guided Fractionation of C. lipii

**Figure 4:**
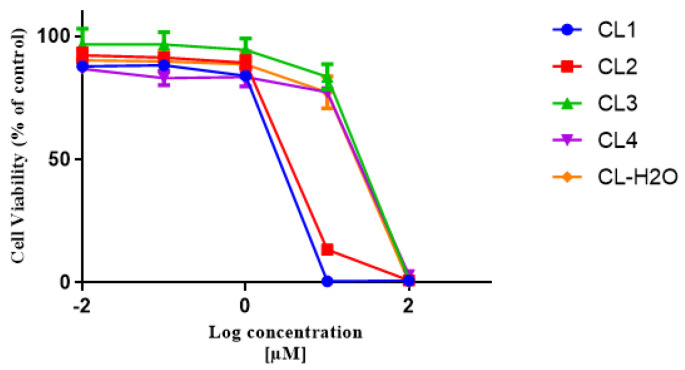
Dose response curves of *C. lipii* fractions towards drug-sensitive parental CCRF-CEM tumor cells.

According to the biological activity against CCRF-CEM cell lines that we previously reported, the methylene chloride/methanol (1: 1) fraction of *C. lipii* showed significant cytotoxic activity against CCRF-CEM with IC_50_ 4.30 µM [9]. Consequently, *C.* lipii extract was fractioned using a flash column to obtain five collective fractions. The cytotoxicity of these subfractions was evaluated against a drug-sensitive CCRF-CEM leukemia cell line. Fraction 1 (CL1) was found to be the most potent cytotoxic fraction with an IC_50_ value 1.81 µM (Figure 4).

## 3. Discussion

In our study, an LC-MS/MS data analysis approach was adopted to highlight the metabolic diversity of Egyptian *Centaurea* species with the aid of molecular networks and the in silico fragmentation trees generated by Sirius. The adopted methodology was advantageous in mapping the chemical space of *Centaurea* species that included cinnamic acids, sesquiterpene lactones, flavonoids, and lignans. Among the annotated features, 21 compounds are reported to occur in the genus *Centaurea* for the first time.

Additionally, the molecular networks delineated the uniqueness of the metabolic profile of *C*. *lipii*, being especially rich in sesquiterpene lactones which might explain its potent cytotoxic activity against multidrug-resistant cancer lines.

For instance, sesquiterpene lactones were detected solely in *C. lipii* (Figure 3) belonging to the germacranolides, guaianolides, cadinanolides, elemanolides, and eudesmanolides. Diverse biological activities were reported for sesquiterpene lactones, including anti-inflammatory, antiparasitic, antiviral, cytotoxic, and others [18]. Moreover, sesquiterpene lactones were recognized as potential candidates for cancer treatment owing to their selective inhibition of tumor and cancer stem cells [18]. Indeed, former investigations have highlighted that the biological activity of sesquiterpene lactones is attributed to the inhibition of enzymes, transcription factors, and/or functional proteins [18].

Since the late 1960s, the cytotoxicity of the sesquiterpene lactones has been investigated to understand the underlying structure–activity relationships. The exocyclic α-methylene-γ-lactone, together with the cyclopentenone and/or α, β-unsaturated ester, has a pivotal role in enhancing cytotoxicity [19]. Further comparisons of different scaffolds revealed that guaianolides and pseudoguaianolides possess the most potent activity [20]. These findings might explain the pronounced cytotoxic activity observed in *C. lipii* in comparison to the other *Centaurea* species.

Bioassay-guided fractionation confirmed such an assumption and led to the isolation of cynaropicrin from the cytotoxic fraction with an IC_50_ of 1.817 µM against the CCRF-CEM leukemia cell line. Cynaropicrin has been formerly reported to exist in several *Centaurea* species, such as *C. behen* [21], *C. ruthenica* [22], and others. Additionally, its cytotoxic activity against the CCRF-CEM leukemia cell line was formerly documented with an IC_50_ value of 0.473 μg/mL [23]. Its cytotoxic properties were correlated to its ability to diminish the generation of intracellular reactive oxygen species involved in carcinogenesis [24].

In conclusion, the described analysis proved efficient and competent for mapping and correlating the constitutive metabolome of the selected *Centaurea* species and simultaneously allowed for the rapid detection of the bioactive metabolites. The outcomes were further validated through bioactivity-guided isolation of the bioactive scaffold.

## 4. Materials and Methods

### 4.1. Plant Materials

Plant samples were collected from their respective locations as listed in Table 1 and were identified by Prof. Dr. Kamal M. Zayed and Prof. Dr. Ibrahim Ahmed Elgarf, taxonomists, Botany Department, Faculty of Science, Cairo University, Egypt. Voucher specimens were deposited in the National Research Center’s herbarium (CAIRC), Department of Phytochemistry and Plant Systematics, with respective voucher numbers as tabulated in Table 1.

### 4.2. Chemicals

#### 4.2.1. Chemicals and Reagents

Methylene chloride, methanol, and acetonitrile were purchased from Sigma Aldrich (Steinheim, Germany). All the solvents used were of HPLC grade.

#### 4.2.2. Preparation of the Extracts

The air-dried powdered aerial parts of the studied *Centaurea* species (100 g each) were macerated separately in 1 L CH_2_Cl_2_/MeOH (1:1) for 24 h at room temperature and then filtered. The filtrates were then evaporated under reduced pressure, lyophilized, and kept frozen at −20 °C for further analyses.

#### 4.2.3. LC-MS/MS Data Acquisition

Dried CH_2_Cl_2_/MeOH (1:1) extract of each species was redissolved in MeOH (HPLC grade) to a final concentration of 2 µg/mL. Chromatographic separation was performed as described before [25].

#### 4.2.4. Data Preprocessing, Molecular Networking, and Compound Dereplication

The feature-based molecular network (FBMN) was built from each species’ HPLC- HRMS/MS data (in positive mode). Firstly, The MSConvert program was used to convert raw data files into 32-bit MzXML files, which were then loaded into Mzmine 2.53 for feature identification [26]. The mgf file from the Mzmine was transferred through WinSCP (https://winscp.net accessed on 12 July 2021) to the Global Natural Products Social Molecular Networking platform (https://gnps.ucsd.edu accessed on 12 July 2021) to create an MN following the online protocol [27]. Subsequently, the constructed molecular network was enhanced with a MolNetEnhancer to boost the chemical structural annotation. For visualization of the resulting MN, Cytoscape (ver. 3.8.2.) was used.

Further data analysis was achieved by importing the mgf output file from Mzmine 2.53 to Sirius + CSI: Finger ID 4.4.29 for the molecular formula prediction (C, H, N, O, S, P) and searching the structure database with 10 ppm *m/z* tolerance using PubChem online database [28].

#### 4.2.5. Cell Culture

The CCRF-CEM leukemia cells were kindly provided by Prof. Axel Sauerbrey (Department of Pediatrics, University of Jena, Jena, Germany) [29]. The cell lines were authenticated using Multiplex Cell Authentication (MCA) based on single-nucleotide polymorphism profiling by Multiplexion GmbH (Heidelberg, Germany) as previously detailed [30]. Those cell lines have been in culture for 14 years.

#### 4.2.6. Resazurin Cytotoxicity Assay

The cytotoxicity of C. *lipii* fractions and the isolated compound was determined by the resazurin reduction assay using a modified protocol previously described [9].

#### 4.2.7. Extraction, Separation, and NMR-Based Structure Elucidation

The air-dried powdered aerial parts of *C. lipii* (100 g) were extracted with CH_2_Cl_2_/MeOH (1:1). The extract (9 g) was then fractionated on a Diaion glass column (6 × 60 cm) and eluted with solvent in a gradient of decreasing polarity starting with (100%) H_2_O followed by a gradient of 20% MeOH, 40% MeOH, 50% MeOH, 60% MeOH, 80% MeOH, and finally washed with 100% MeOH. We collected 31 fractions (500 mL of each solvent mixture) based on the thin-layer chromatography profile using a vanillin–sulphuric acid spray reagent for detection. Similar fractions were added to each other based on their chromatographic patterns to yield the final five collective fractions which were H_2_O fraction (0.9 gm), CL-1 (2 gm), CL-2 (1.5 gm), CL-3 (1.2 gm), and CL-4 (2.5 gm). Fraction CL-1 (the most active cytotoxic fraction) was subjected to isolation and purification by HPLC (4.6 × 250 cm) using MeOH: H2O (40: 60%, 2.5 L) with the addition of 1 mL formic acid to afford compound **1** (4.5 mg).

High-performance liquid chromatography (HPLC) was performed on an Agilent pump equipped with an Agilent-G1314 variable wavelength UV detector at 254 nm and a semi-preparative reverse-phase column (Econosphere™, RP-C18, 5 μm, 250 × 4.6 mm, Alltech, Deerfield, IL, USA). Precoated silica gel plates (Kiesel gel 60 F254, 0.25 mm) were used for TLC analyses.

NMR spectra were measured on a Bruker 500 NMR spectrometer (USA) (500 MHz for 1H and 125 MHz for 13C). All chemical shifts (δ) are given in ppm units with reference to TMS as an internal standard, and coupling constants (J) are reported in Hz.

## Figures and Tables

**Table 1:** The studied *Centaurea* species, and their respective collection sites.

Species	Sample Code	Voucher ID	Collection Site	Latitude (N)	Longitude (E)
*C. alexandrina*	Ce.Alex	M/2282	Marsa Matrouh	31°23′37.81″	27°01′7.64″
*C. calcitrapa*	Ce.Co	M/2279	Marsa Matrouh	31°03′41.10″	28°12′31.6″
*C. eryngioides*	CE	M/2284	Saint Catherine	28°33′20.83″	33°56′9.13″
*C. glomerata*	Ce.G	M/2280	Rashid	30°56′52.51″	30°58′33.1″
*C. lipii*	CL	M/2281	Egyptian north coast	29°38′16.55″	32°18′23.72″
*C. pallescens*	Ce.PA	M/2283	Marsa Matrouh	31°22′37.01″	31°03′41.16″
*C. pumilio*	CP	M/2285	Egyptian north coast	30°54′9.06″	29°26′8.63″
*C. scoparia*	Ce.Sco	M/2278	Red Sea Coast	31°03′41.16″	31°03′41.16″

## Data Availability

The created molecular network and parameters can be accessed via the link: https://gnps.ucsd.edu/ProteoSAFe/status.jsp?task=b47430d7801f42eaa9089739417f3aa1 accessed on 21 July 2021.

## Supplementary Materials

The following supporting information can be downloaded at: https://www.mdpi.com/article/10.3390/molecules28020674/s1, Supplementary Figure S1: Overlaid base peak chromatogram of the studied *Centaurea* species in the positive ionization mode; Supplementary Table S1: Compound assignment of the studied *Centaurea* species extracts as revealed by UPLC-HRMS/MS analysis. Reference Citations of [11,31,32,33,34,35,36,37,38,39,40,41,42,43,44,45,46,47,48,49,50,51,52,53,54,55,56,57,58,59,60,61,62,63,64,65,66,67,68,69,70,71,72,73,74,75,76,77,78,79,80].
