# Supplementary material for: Feature-Based Molecular Networking for the Exploration of the Metabolome Diversity of Common Egyptian Centaurea Species in Relation to Their Cytotoxic Activity
Source: Molecules. 2023 Jan 9;28(2):674. doi: 10.3390/molecules28020674 (PMC9862008; doi:10.3390/molecules28020674)
Supplement: Supplementary file 1 [file molecules-28-00674-s001.zip › molecules-2118901-Supplementary.pdf]

Feature-Based Molecular Networking for the Exploration of the Metabolome Diversity of Common Egyptian *Centaurea* Species in Relation to Their Cytotoxic Activity

Eman H. Reda [1], Nesrine M. Hegazi [2], Mona Marzouk [2], Zienab T. Abdel Shakour [1], Ali M. El-Halawany [3], El-Sayeda A. El-Kashoury [3], Tarik A. Mohamed [4], Mahmoud A. A. Ibrahim [5,6], Khaled A. Shams [4], Nahla S. Abdel-Azim [4], Christopher J. Kampf [7], Thomas Efferth [8,*], Paul. W. Paré [9] and Mohamed-Elamir F. Hegazy [4,*]

[1] Phytochemistry Laboratory, National Organization for Drug Control and Research, Giza 12622, Egypt
[2] Phytochemistry and Plant Systematics Department, National Research Centre, Dokki, Cairo 12622, Egypt
[3] Department of Pharmacognosy, Faculty of Pharmacy, Cairo University, Cairo 11562, Egypt
[4] Chemistry of Medicinal Plants Department, National Research Centre, 33 El-Bohouth St., Dokki, Giza 12622, Egypt
[5] Computational Chemistry Laboratory, Chemistry Department, Faculty of Science, Minia University, Minia 61519, Egypt[6] School of Health Sciences, University of Kwa-Zulu-Natal, Westville, Durban 4000, South Africa
[7] Department for Chemistry, Johannes Gutenberg University Mainz, Duesbergweg 10-14, 55128 Mainz, Germany
[8] Department of Pharmaceutical Biology, Institute of Pharmaceutical and Biomedical Sciences Chemistry, Johannes Gutenberg University, Staudinger Weg 5, 55128 Mainz, Germany
[9] Department of Chemistry & Biochemistry, Texas Tech University, Lubbock, TX 79409, USA
* Correspondence: efferth@uni-mainz.de (T.E.); mohegazy@uni-mainz.de (M.-E.F.H.)

Supplementary Data

Supplementary Figure S1: Overlaid base peak chromatogram of the studied Centaurea species in the positive ionization mode.

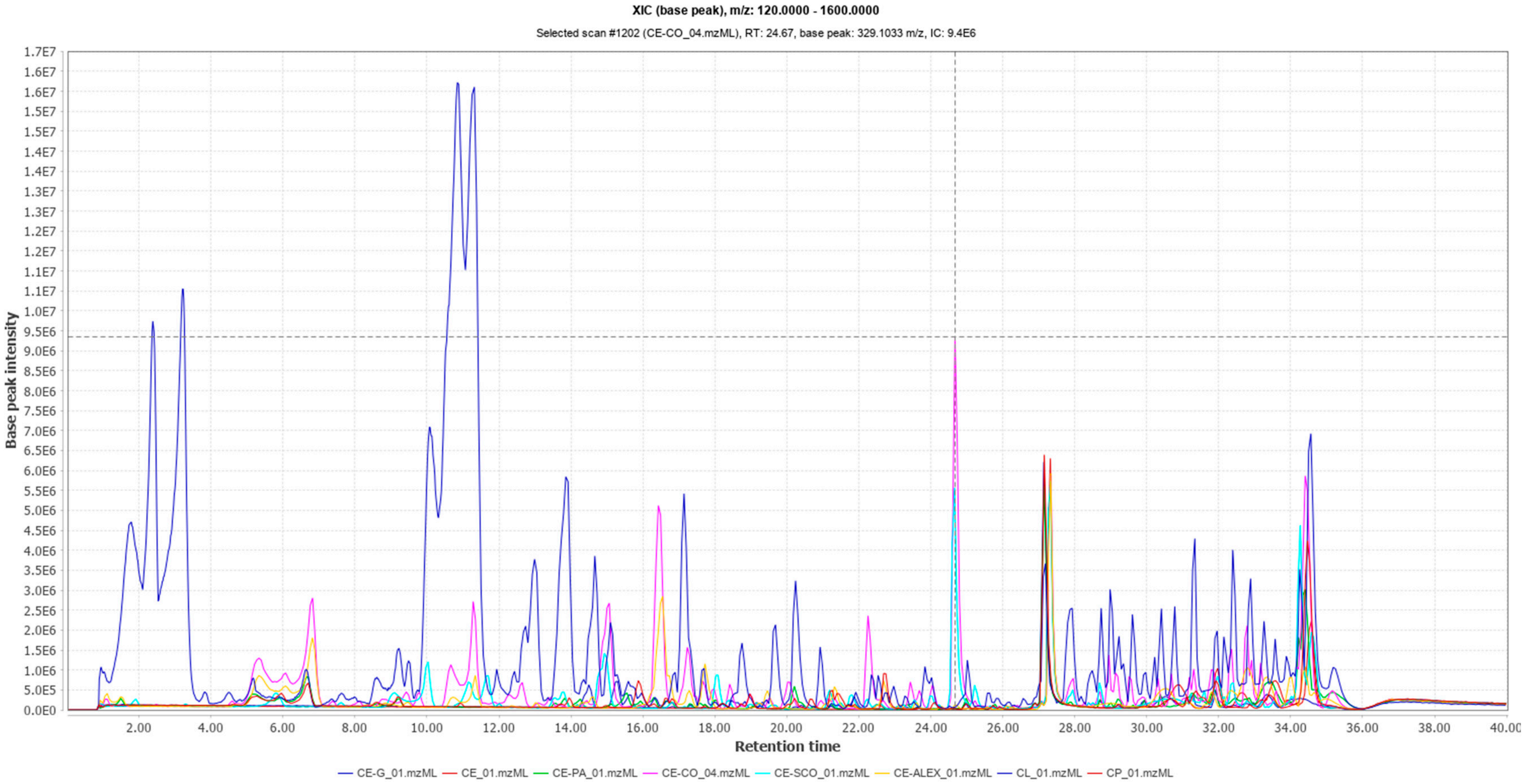

Supplementary Table S1: Compound assignment of the studied *Centaurea* species extracts as revealed by UPLC-HRMS/MS analysis. Ce: *C. eryngioides* **Ca:** *C. alexandrina,* **CC:** *C. calcitrapa,* **Cg:** *C. glomerata,* **Cpa:** *C. pallescens,* **Cs:** *C. scoparia,* **Cl:** *C. lipii,* and **Cpu:** *C. pumilio.*

No.	Class	RT	*m/z*, adduct	MS^2	Compound name	Molecular formula, error (ppm)	References	Ce	Ca	Cc	Cg	Cpa	Cs	Cl	Cpu
**1**	Cinnamic acid ester	8.97	369.1179 $[M+H]^+$	177, 145, 117	Feruloylquinic acid [a]	$C_{19}H_{20}O_9$ (0.59)	11, GNPS libraries	-	-	+	+	-	+	+	-
**2**	Germacranolide sesquiterpene lactone glycoside	9.7	446.2385 $[M+NH_4]^+$	231, 203, 185, 175, 157, 147	Dihydroparthenolide -*O*- hexoside isomer I [b]	$C_{21}H_{32}O_9$ 0.08	31	+	-	+	-	-	-	-	-
**3**	Coumarin	10.33	193.0499 $[M+H]^+$	161, 150	Scopoletin/ isoscopoletin [a]	$C_{10}H_8O_4$ (0.44)	32,33	-	+	-	-	-	-	-	-
**4**	Coumarin	10.65	193.0497 $[M+H]^+$	161, 150	Scopoletin/ isoscopoletin [a]	$C_{10}H_8O_4$ (0.88)	32,33	-	+	-	-	-	-	-	-
**5**	Germacranolide sesquiterpene lactone	11.07	436.1966 $[M+C_2H_3N+H]^+$	102, 74, 56	7-hydroxy-10-(hydroxymethyl)-6-methyl-3-methylidene-2-oxo--cyclodeca[b]furan-4-y-3,4-dihydroxy-2-methylidenebutanoate [a]	$C_{20}H_{26}O_8$ (0.84)	34	-	-	-	-	-	-	+	-

**6**	Germacranolide sesquiterpene lactone glycoside	11.37	446.2380 $[M+NH_4]^+$	231, 203, 185, 175, 157, 147	Dihydroparthenolide -*O*- hexoside isomer II [b]	$C_{21}H_{32}O_9$ (0.08)	31	+	-	+	-	-	-	-	-
**7**	Germacranolide esesquiterpene lactone	11.56	464.2252 $[M+ C_2H_3N +H]^+$	316, 128, 102, 84	10-(hydroxymethyl)-3,6-dimethyl-2-oxo-cyclodeca[b]furan-4-yl -4-(acetyloxy)-2-(hydroxymethyl)but-2-enoate [a]	$C_{22}H_{30}O_8$ (0.22)	35	-	-	-	-	-	-	+	-
**8**	Cadinanolides sesquiterpene lactone	11.63	382.2220 $[M+ C_2H_3N+H]^+$	336, 296, 296, 268, 250, 232, 130, 102, 84	acetoxy-dihydroxy-tetr ahydro-artemisinic acid methyl ester [b]	$C_{18}H_{28}O_6$ (0.05)	36	-	+	+	+	+	+	+	+
**9**	Flavonoid-*O*-glycoside	11.71	671.18 $[M+H]^+$	347, 332, 197, 193, 145	Spinacetin-*O*-gentiobioside [b]	$C_{29}H_{34}O_{18}$ (0.07)	37	-	+	-	-	+	-	-	-
**10**	Germacranolide esesquiterpene lactone	12.04	462.1227 $[M+ C_2H_3N +H]^+$	220, 174, 128	4-acetylcnicin [a]	$C_{22}H_{28}O_8$ (1.00)	38	-	-	-	-	-	-	+	-

**11**	Germacranolide sesquiterpene lactone	12.58	496.2529 [M+ C_2H_3N +H]+	450, 318, 130, 102, 84	incaspitolide D [b]	$C_{23}H_{34}O_9$ (-0.53)	39	-	-	+	-	-	-	-	-
**12**	Flavonoid -*C*-glycoside	13.80	433.1135 [M+H]+	313, 283	(iso)vitexin [a]	$C_{21}H_{20}O_{10}$ (0.01)	40	-	-	-	+	+	-	+	-
**13**	Flavonoid -*O*-glycoside	13.83	495.1129 [M+H]+	333, 318	Patuletin-*O*-glucoside, isomer I [a]	$C_{22}H_{22}O_{13}$ (0.25)	41	-	+	-	+	+	-	+	+
**14**	Lignans	13.97	538.2286 [M+NH_4]+	359, 341, 323, 291, 163, 137	Matairesinol -*O*-glucoside [a]	$C_{26}H_{32}O_{11}$ (0.25)	42	-	+	-	-	-	-	+	+
**15**	Flavonoid -*O*-glycoside	14.30	495.1133 [M+H]+	333, 318, 287	Patuletin -*O*-glucoside isomer II [a]	$C_{22}H_{22}O_{13}$ (0.04)	43	-	+	-	-	+	+	-	-
**16**	Flavonoid -*O*-glycoside	14.58	595.1663 [M+H]+	287, 205	Luteolin-7-*O*-rutinoside [a]	$C_{27}H_{30}O_{15}$ (0.86)	44	+	+	+	-	-	-	-	-
**17**	Flavonoid -*O*-glycoside	14.60	495.1137 [M+H]+	333, 318	Patuletin-*O*-glucoside, isomer III [a]	$C_{22}H_{22}O_{13}$ (0.48)	41	-	+	+	+	+	+	-	-
**18**	Flavonoid -*O*-glycoside	14.65	449.1082 [M+H]+	432, 287, 269	Luteolin-7-*O*-glucoside [a]	$C_{21}H_{20}O_{11}$ (-0.94)	45	-	+	+	+	-	+	+	-
**19**	Flavonoid -*O*-glycoside	14.69	479.1185 [M+H]+	317, 302,	Isorhamnetin -*O*-glucoside [a]	$C_{22}H_{22}O_{12}$ (-0.07)	46	-	-	+	+	+	+	+	-

				257, 165												
**20**	Cinnamic acid amides	14.73	314.1388 $[M+H]^+$	177, 145, 121	*N*-Feruloyl tyramine isomer I [b]	$C_{18}H_{19}O_4$ (-1.56)	47, GNPS	+	+	+	-	-	+	-	+
**21**	Lignans	14.84	552.2282 $[M+NH_4]^+$	373, 355, 337, 323, 305, 295, 237, 219, 201, 137	Arctiin / Arctigenin-*O*-glucoside isomer I [a]	$C_{27}H_{34}O_{11}$ (0.09)	42,48	-	+	-	-	-	+	-	+
**22**	Guaianolides sesquiterpene lactone	14.85	472.2333 $[M+C_2H_3N+H]^+$	228, 210, 182, 128	Daucoguaianolactone F [b]	$C_{24}H_{30}O_7$ (0.24)	49	-	-	-	-	-	-	+	-
**23**	Guaianolides sesquiterpene lactone	14.89	432.2012 $[M+ C_2H_3N +H]^+$	227, 209, 199, 183, 98	8-(acetyloxy)-9-hydroxy-9-(hydroxymethyl)-3,6-dimethylidene-2-oxo-octahydroazuleno[4,5-b]furan-4-yl 2-(hydroxymethyl)prop-2-enoate [a]	$C_{21}H_{26}O_7$ (-0.11)	50	-	-	-	-	-	-	+	-
**24**	Flavonoid -*O*-glycoside	14.99	477.1029 $[M+H]^+$	301, 286	Hispidulin-7-*O*-glucuronide isomer I [a]	$C_{22}H_{20}O_{12}$ (-1.11)	51	-	+	-	+	+	-	+	+

**25**	Flavonoid -*O*-glycoside	15.14	509.1289 [M+H]+	347, 332, 317	Spinacetin -*O*-glucoside isomer I [a]	$C_{23}H_{24}O_{13}$ (-1.48)	52	-	+	-	-	+	-	-	-
**26**	Flavonoid -*O*-glycoside	15.23	581.1502 [M+H]+	287, 269	Luteolin-*O*- pentosyl – hexoside [b]	$C_{26}H_{28}O_{15}$ (-0.23)	Öztürk et al., 2019	-	+	-	-	-	+	-	-
**27**	Flavonoid -*O*-glycoside	15.39	479.1185 [M+H]+	317, 302, 274, 229	Isorhamnetin -*O*-glucoside isomer I [a]	$C_{22}H_{22}O_{12}$ (-0.7)	46	-	+	-	+	+	-	+	+
**28**	Lignans	15.52	582.2568 [M+NH4]+	385, 353, 267, 207	Acetyl matairesinoside, Yunnanensin B [b]	$C_{28}H_{36}O_{12}$ (0.86)	53	-	-	-	-	-	-	+	-
**29**	Lignans	15.62	552.2438 [M+NH4]+	373, 355, 337, 323, 305, 295, 237, 219, 201, 137	Arctiin / Arctigenin-*O*-glucoside isomer II [a]	$C_{27}H_{34}O_{11}$ (0.09)	42,48	-	+	+	+	+	-	+	+
**30**	Flavonoid -*O*-glycoside	15.71	477.1029 [M+H]+	301, 286	Hispidulin-*O*-glucuronide isomer II [a]	$C_{22}H_{20}O_{12}$ (-1.11)	51	-	-	-	-	-	-	-	+

**31**	Flavonoid -*O*-glycoside	15.77	447.0927 $[M+H]^+$	271	Apigenin -*O*-glucuronide isomer I [a]	$C_{21}H_{18}O_{11}$ (-0.6)	51	-	-	+	-	+	-	+	+
**32**	Lignans	15.81	327.1594 $[M+H]^+$	163, 137	Secoisolariciresinol [b]	$C_{20}H_{22}O_4$ (0.84)	54	-	+	+	-	+	+	+	-
**33**	Flavonoid -*O*-glycoside	15.87	447.0934 $[M+H]^+$	271	Apigenin -*O*-glucuronide isomer II [a]	$C_{21}H_{18}O_{11}$ (2.74)	51	+	+	+	+	+	-	+	+
**34**	Flavonoid -*O*-glycoside	15.90	463.1240 $[M+H]^+$	301, 286	monomethoxy trihydroxyflavone -*O*-glucoside isomer I [a]	$C_{22}H_{22}O_{11}$ (-0.95)	55	-	+	-	+	+	-	+	+
**35**	Cinnamic acid amides	15.95	314.1385 $[M+H]^+$	177, 145, 121	*N*-feruloyl tyramine isomer II [b]	$C_{18}H_{19}NO_4$ (0.15)	47, GNPS libraries	-	-	-	+	+	-	+	+
**36**	Flavonoid -*O*-glycoside	16.18	433.1132 $[M+H]^+$	271	Apigenin-*O*-hexoside [a]	$C_{21}H_{20}O_{10}$ (-0.51)		-	+	+	-	-	-	-	-
**37**	Flavonoid -*O*-glycoside	16.38	479.1191 $[M+H]^+$	317, 302, 257, 165	Isorhamnetin -*O*-glucoside isomer II [a]	$C_{22}H_{22}O_{12}$ (-0.11)	46	-	+	-	-	-	-	-	-
**38**	Guaianolides sesquiterpene lactone glycoside	16.38	464.1936 $[M+C2H3N+H]^+$	220, 202, 181	Dehydrolactuside C [b]	$C_{21}H_{26}O_9$ (3.19)	56	-	-	-	-	-	-	+	-
**39**	Lignans	16.46	552.2438 $[M+NH_4]^+$	373, 355, 337,	Arctiin / Arctigenin-*O*-glucoside isomer III [a]	$C_{27}H_{34}O_{11}$ (0.09)	42,48	-	+	-	-	-	-	-	-

				323, 305, 295, 237, 219, 201, 137											
**40**	Flavonoid -*O*-glycoside	16.67	509.1298 [M+H]+	347	Spinacetin -*O*-glucoside isomer II [a]	$C_{23}H_{24}O_{13}$ (-1.6)	52	+	+	+	+	+	+	-	-
**41**	Flavonoid -*O*-glycoside	16.73	493.1342 [M+H]+	331, 316	Trihydroxy-dimethoxy-flavone –*O*-glucoside isomer I /Jaceoside [a]	$C_{23}H_{24}O_{12}$ (-0.96)	57	-	-	+	+	+	+	+	+
**42**	Flavonoid -*O*-glycoside	16.84	463.1235 [M+H]+	301, 286, 255	monomethoxy trihydroxyflavone -*O*-glucoside isomer II [a]	$C_{22}H_{22}O_{11}$ (-1.59)	58	-	+	+	+	+	+	-	-
**43**	Flavonoid -*O*-glycoside	16.86	523.1451 [M+H]+	361, 346, 328	Trimethoxy trihydroxyflavone-*O*-glucoside isomer I (Centaurein) [a]	$C_{24}H_{26}O_{13}$ (0.3)	59	-	-	-	-	+	-	-	-
**44**	Germacranolidesesquiterpene lactone	17.33	480.2583 [M+ C_2H_3N +H]+	434, 318, 130, 102, 84	incaspitolide A [b]	$C_{23}H_{34}O_8$ (-0.2)	60	-	-	+	-	-	-	-	-
**45**	Acylated flavonoid -*O*-glycoside	17.57	537.1239 [M+H]+	333, 318, 301,	Pentahydroxy -monomethoxy flavone -*O*-acetyl	$C_{24}H_{24}O_{14}$ (-0.05)	GNPS libraries	-	-	-	t	+	-	-	-

				273	hexoside [b]										
**46**	Elemanolide sesquiterpene lactone glycosides	17.6	560.2703 [M+NH4]+	249, 231, 203, 185, 175, 157	sarcaglaboside D [b]	$C_{26}H_{38}O_{12}$ (-0.12)	61	-	+	-	-	-	-	-	-
**47**	Guaianolide sesquiterpene lactone	17.64	432.2023 [M+H]+	330, 286, 312, 268, 244, 70	Clementein [a]	$C_{21}H_{26}O_{7}$ (0.94)	62	-	-	-	-	-	-	+	-
**48**	Flavonoid -*O*-glycoside	17.66	493.1341 [M+H]+	331, 316	Trihydroxy-dimethoxy-flavone – *O*-glucoside isomer II /Jaceoside [a]	$C_{23}H_{24}O_{12}$ (-0.52)	57	-	+	-	-	+	+	-	-
**49**	Flavonoid -*O*-glycoside	17.69	523.1450 [M+H]+	361, 346, 328	Trimethoxy trihydroxyflavone-*O*-glucoside isomer II (Jacein) [a]	$C_{24}H_{26}O_{13}$ (-1.67)	59	-	+	-	-	+	-	-	-
**50**	Lignans	17.75	359.1496 [M+H]+	291, 263, 249	Matairesinol [a]	$C_{20}H_{22}O_{6}$ (-0.77)	63	-	+	+	-	+	+	-	+
**51**	Acylated flavonoid -*O*-glycoside	17.83	607.1670 [M+H]+	301	Rhamnocitrin-*O*-hydroxy-methylglutaryl-hexoside [b]	$C_{28}H_{30}O_{15}$ (2.3)	64	-	-	-	-	-	-	+	-
**52**	Acylated	18.29	461.1077	271	Apigenin *O*-methyl	$C_{22}H_{20}O_{11}$	65	-	-	-	+	+	-	+	+

	flavonoid -*O*-glycoside		[M+H]$^+$		glucuronide [a]	(0.62)									
**53**	Lignans	18.49	373.1637 [M+H]$^+$	305, 291, 274, 246, 177, 151, 137	[(dimethoxyphenyl) methyl]-3- [(hydroxy-methoxyphenyl) methyl]-tetrahydrofuranone/ Arctigenin [a]	$C_{21}H_{24}O_6$ (0.39)	63	-	+	-	+	+	+	+	+
**54**	Flavonoids	18.74	317.0657 [M+H]$^+$	302, 274, 257	Quercetin methyl ether isomer I [a]	$C_{16}H_{12}O_7$ (1.16)	66	-	+	+	+	+	+	+	+
**55**	Lignans	18.98	390.1914 [M+NH$_4$]$^+$	291, 235, 177, 151, 137	[(dimethoxyphenyl) methyl]-3- [(hydroxy-methoxyphenyl) methyl]-tetrahydrofuranone [a]	$C_{21}H_{27}O_6$ (-1.11)	63	-	-	-	+	+	-	+	+
**56**	Acylated flavonoid -*O*-glycoside	19.01	491.1192 [M+H]$^+$	301, 286	Luteolin-*O*-acetyl hexoside [b]	$C_{23}H_{22}O_{12}$ (1.06)	67	-	-	-	-	-	-	-	+
**57**	Acylated flavonoid -*O*-glycoside	19.15	521.1291 [M+H]$^+$	317, 302, 274	Isorhamnetin -*O*-acetyl hexoside [b]	$C_{24}H_{24}O_{13}$ (0.79)	68, GNPS libraries	-	+	+	+	+	+	+	-
**58**	Acylated flavonoid -*O*-glycoside	19.12	551.1389 [M+H]$^+$	347, 332	Syringetin *O*-acetyl hexoside [b]	$C_{25}H_{26}O_{14}$ (-0.94)	69	-	-	-	+	-	-	-	-

**59**	Lignans	19.38	373.1651 [M+H]+	291, 235, 177, 151, 137	[(dimethoxyphenyl) methyl]-3- [(hydroxy-methoxyphenyl) methyl]-tetrahydrofuranone / Arctigenin (Isomer II) [a]	$C_{21}H_{24}O_6$ (-1.65)	63	-	+	+	+	+	+	+	-
**60**	Flavonoids	19.67	287.0550 [M+H]+	269, 241, 153, 135	Luteolin [a]	$C_{15}H_{10}O_6$ (0.42)	70 GNPS libraries	-	+	-	-	-	-	-	+
**61**	Flavonoids	19.86	317.0658 [M+H]+	302, 274, 257	quercetin mehyl ether [a]	$C_{16}H_{12}O_7$ (-0.9)	71	-	+	-	+	+	+	+	+
**62**	Flavonoids	20.02	375.1079 [M+H]+	359, 342, 317, 299	Quercetagetin tetramethyl ether [a]	$C_{19}H_{18}O_8$ (0.26)	72	-	-	-	-	-	-	+	+
**63**	Flavonoids	20.06	331.0819 [M+H]+	316, 301, 275	Trihydroxy-dimethoxyflavone isomer II [a]	$C_{17}H_{14}O_7$ (-0.19)	73,74	-	-	-	-	-	-	+	+
**64**	Flavonoids	20.27	301.0705 [M+H]+	286, 258, 168	Trihydroxy-monomethoxyflavone (Isomer I) / Hispidulin [a]	$C_{16}H_{12}O_6$ (0.44)	51	+	+	+	+	+	+	+	+

**65**	Flavonoids	20.33	361.0918 [M+H]+	346, 331, 303, 269	Trihydroxy-trimethoxyflavone (Isomer I) isomer [a]	$C_{18}H_{16}O_8$ (-0.44)	43,75, GNPS libraries	-	+	+	+	+	-	+	+
**66**	Cinnamic acid amides	20.37	337.1563 [M+H]+	177, 145, 117	*N*-feruloyl tryptamine [b]	$C_{20}H_{20}N_2O_3$ (2.7)	47	-	+	-	-	-	-	-	-
**67**	Flavonoids	20.81	317.0657 [M+H]+	302, 274	Quercetin methyl ether isomer II [a]	$C_{16}H_{12}O_7$ (1.06)	66	-	+	-	-	-	-	-	-
**68**	Flavonoids	20.94	361.0917 [M+H]+	346, 331, 303, 269	Trihydroxy-trimethoxyflavone isomer II [a]	$C_{18}H_{16}O_8$ (1.09)	43	-	+	+	+	-	-	+	+
**69**	Flavonoids	21.16	301.0711 [M+H]+	286, 258, 168	Trihydroxy-monomethoxyflavone isomer II [a]	$C_{16}H_{12}O_6$ (-1.44)	76	-	+	+	+	+	-	+	+
**70**	Flavonoids	21.18	271.0605 [M+H]+	153, 119, 109	Apigenin [a]	$C_{15}H_{10}O_5$ (-1.34)	70 GNPS libraries	-	+	-	-	-	+	-	+
**71**	Flavonoids	21.28	331.0819 [M+H]+	316, 301, 273, 245	Trihydroxy-dimethoxyflavone isomer III [a]	$C_{17}H_{14}O_7$ (-0.26)	77	-	+	+	+	+	+	+	+
**72**	Eudesmanolide	21.42	446.2173 [M+ C_2H_3N	330, 286,	3-propyloxy-6-methyl-	$C_{22}H_{28}O_7$ (0.03)	78	-	-	-	-	-	-	+	-

	sesquiterpene lactone		+H]+	312, 70	cryloxyivangustin [b]										
**73**	Flavonoids	21.45	315.0864 [M+H]+	300, 257, 168	Dihydroxy-dimethoxyflavone (Isomer I) [a]	$C_{17}H_{14}O_6$ (-1.58)	77	-	+	+	+	-	+	+	+
**74**	Flavonoids	21.92	361.0919 [M+H]+	346, 331, 303, 269	Trihydroxy-trimethoxyflavone isomer III [a]	$C_{18}H_{16}O_8$ (-0.28)	43,45	-	+	-	-	+	-	-	-
**75**	Flavonoids	21.93	345.0969 [M+H]+	330, 315, 284, 169	Dihydroxy-trimethoxy flavone [a]	$C_{18}H_{16}O_7$ (0.5)	79	+	+	+	+	+	+	+	+
**76**	Flavonoids	22.29	315.0865 [M+H]+	300, 257, 168	Dihydroxy-dimethoxyflavone isomer II [a]	$C_{17}H_{14}O_6$ (-1.83)	77	+	+	+	-	+	+	-	+
**77**	Flavonoids	23.36	315.0866 [M+H]+	300, 284, 257, 168	Dihydroxy-dimethoxyflavone isomer III [a]	$C_{17}H_{14}O_6$ (0.87)	77	+	+	+	+	+	+	+	+
**78**	Flavonoids	23.61	315.0864 [M+H]+	300, 272, 257, 168	Dihydroxy-dimethoxyflavone isomer VI [a]	$C_{17}H_{14}O_6$ (1.82)	77	+	-	+	-	-	+	-	-
**79**	Flavonoids	24.05	315.0866 [M+H]+	300, 257	Dihydroxy-dimethoxyflavone isomer V [a]	$C_{17}H_{14}O_6$ (-1.84)	77	+	-	+	-	-	+	-	-

**80**	Flavonoids	24.09	329.103 [M+H]+	314, 296, 268, 135	Monohydroxy-trimethoxyflavone isomer I [a]	$C_{18}H_{16}O_6$ (-2.4)	80	+	+	+	+	+	+	-	
**81**	Flavonoids	24.70	329.1026 [M+H]+	314, 296, 268, 135	Monohydroxy-trimethoxyflavone isomer II [a]	$C_{18}H_{16}O_6$ (-0.01)	80	+	-	+	-	-	+	-	+

[a,] Compounds previously isolated from *Centaurea* species

[b] Compounds first time to be reported in *Centaurea* species
